# Primary idiopathic chylopericardium: a retrospective case series

**DOI:** 10.1186/s12893-015-0047-8

**Published:** 2015-05-12

**Authors:** Zhijun Han, Shanqing Li, Hongli Jing, Hongsheng Liu

**Affiliations:** Department of Thoracic Surgery, Peking Union Medical College Hospital (PUMCH), Chinese Academy of Medical Sciences & Peking Union Medical College (CAMS & PUMC), 1 Shuai-Fu-Yuan, Beijing, 100730 China; Department of nuclear medicine, Peking Union Medical College Hospital (PUMCH), Chinese Academy of Medical Sciences & Peking Union Medical College (CAMS & PUMC), 1 Shuai-Fu-Yuan, Beijing, 100730 China

**Keywords:** Pericardial effusion, Chyle, Diagnostic techniques and procedures, Drug therapy, Thoracic surgery

## Abstract

**Background:**

Primary idiopathic chylopericardium is a rare clinical entity characterized by the accumulation of chyle within the pericardial cavity without a definitive cause. The aim of this study was to assess the clinical presentation, etiology, diagnosis, treatment and follow-up of primary idiopathic chylopericardium.

**Methods:**

We retrospectively reviewed 9 cases of patients who suffered from primary idiopathic chylopericardium at our hospital from January 1993 to November 2013.

**Results:**

There were two males and seven females among our patients. Their ages ranged from 13 to 55 years. The most common clinical presentation was dyspnea. The etiology was idiopathic. All patients were diagnosed by pericardiocentesis, computed tomography of the chest and lymphoscintigraphy. Non-surgical therapy was adopted in all nine patients and failed in six, who underwent subsequent successful surgery. Thoracic duct ligation with the creation of a pericardial window was the most common surgical procedure. All patients were followed up from 3 months to 9 years, and no recurrence occurred.

**Conclusions:**

In assessing patients with an enlarged cardiac silhouette, one should be aware of primary idiopathic chylopericardium. The most effective treatment is ligation of the thoracic duct and the creation of a pericardial window.

## Background

The accumulation of chyle in the pericardial space, or chylopericardium, is a condition that occurs most frequently after trauma, cardiothoracic surgery, or radiation therapy or in association with tumors or tuberculosis. Congenital lymphangiomatosis or lymphangiectasia may also be a cause of chylopericardium [1,2]. However, if the precise etiology cannot be identified, the condition is called primary or idiopathic chylopericardium, which is a rare clinical entity. Herein, we report 9 cases of primary idiopathic chylopericardium and assess the clinical presentation, etiology, diagnosis, treatment and follow-up of this entity. A review of the literature is also presented.

## Methods

There were ten patients diagnosed with primary idiopathic chylopericardium at our hospital between January 1993 and November 2013. One patient was excluded due to insufficient data. We retrospectively reviewed the clinical presentation, etiology, diagnosis, treatment and follow-up of the remaining nine patients. The Ethics Review Committee of Peking Union Medical College Hospital approved this retrospective study. The patient records/information used in this study were anonymized and de-identified prior to analysis.

## Results

The characteristics of the nine patients with primary idiopathic chylopericardium are summarized in the Table 1.

**Table 1:** The characteristics of the nine patients with primary idiopathic chylopericardium

Case	Year	Symptoms	Lymphoscintigraphy	Non-surgical treatment	Surgical treatment	Complications	Follow-up
1	2000	Dyspnea	Communication	Pericardiocentesis, MCT diet	No	No	3 M
2	2002	Dyspnea	Communication	Pericardiocentesis, MCT diet	Right thoracotomy, L + W	Chylothorax; religation of the thoracic duct	1 Y
3	2004	Dyspnea	Communication	Pericardiocentesis, MCT diet, pericardiostomy	No	No	9 Y
4	2004	Atrial fibrillation	Pericardial accumulation	Pericardiocentesis, MCT diet	Right thoracotomy, L + W	No	6 M
5	2006	Dyspnea	Pericardial accumulation	Pericardiocentesis, MCT diet	Midsternal thoracotomy, L + W	No	1 Y
6	2006	Dyspnea	Pericardial accumulation	Pericardiocentesis, MCT diet	No	No	9 M
7	2008	Dyspnea	Negative	Pericardiocentesis, MCT diet	Right thoracotomy, L + W	No	2 Y
8	2008	Asymptomatic	Negative	Pericardiocentesis, MCT diet	Right thoracotomy, L + W	No	8 M
9	2009	Asymptomatic	Negative	Pericardiocentesis, MCT diet	Right VATS, L + W	No	3 Y

*M* months, *Y* years, *MCT* medium chain triglyceride, *VATS* video-assisted thoracic surgery, *L* ligation of the thoracic duct, *W* pericardial window formation, *Communication* communication between the thoracic duct and the pericardial space, *Pericardial accumulation* pericardial accumulation of technetium-99 m sulfur colloid

### Clinical presentation and diagnosis

**Fig. 1:**
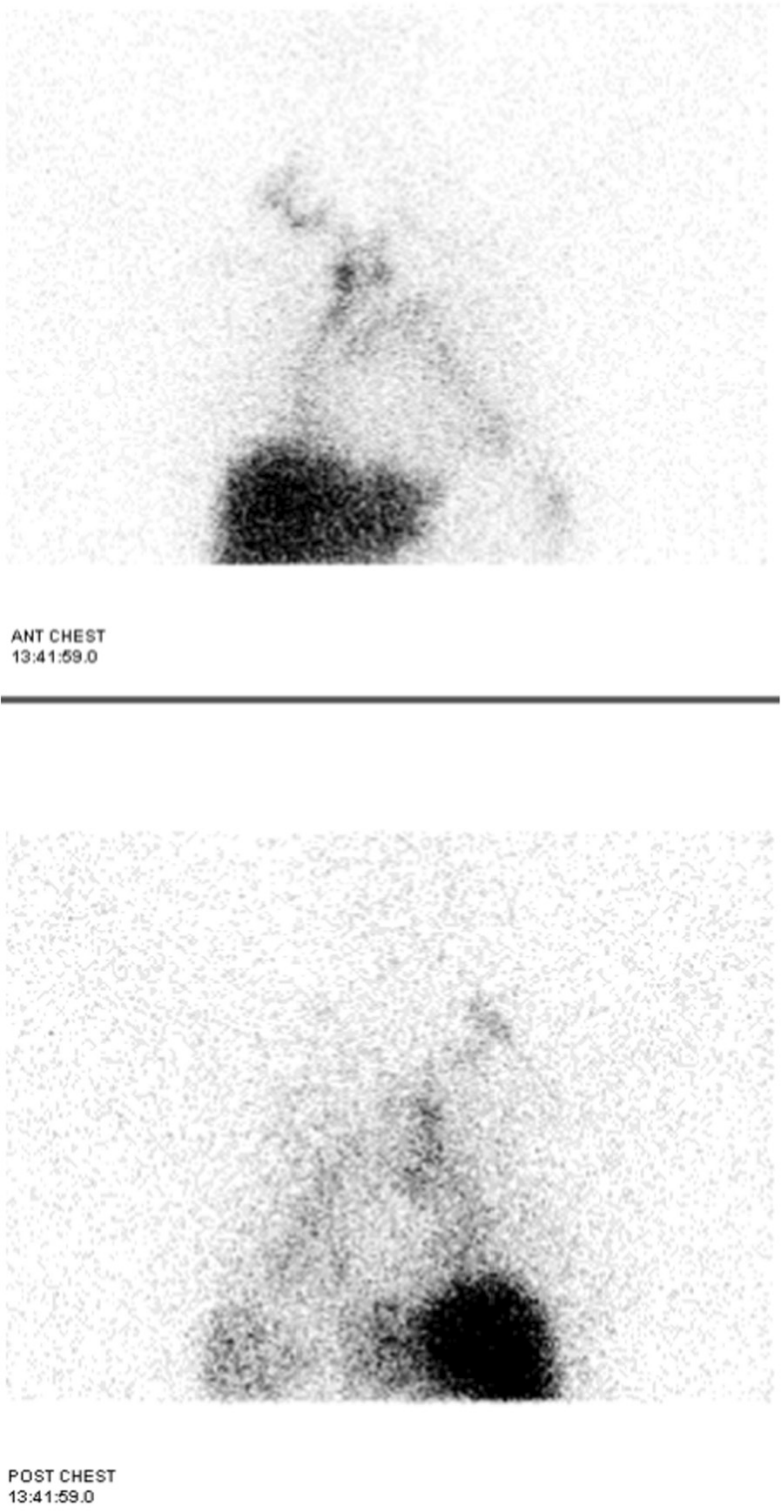
Lymphoscintigraphy. The local images were collected after injecting Tc-99 m antimony sulfide colloid 5.5 hours. The markedly increased activity in the areas of the pericardium was seen

**Fig. 2:**
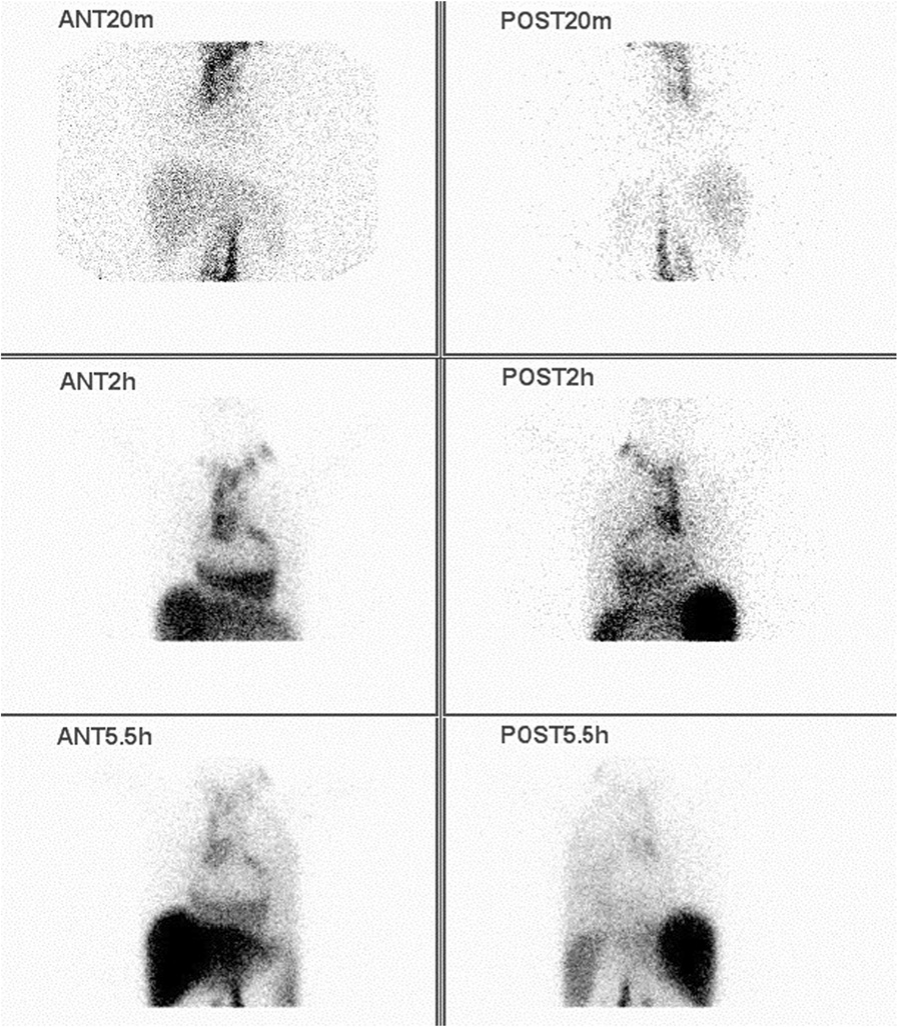
Lymphoscintigraphy. The local images were collected after injecting Tc-99 m antimony sulfide colloid 20 minutes, 2 and 5.5 hours. Lymphoscintigraphy demonstrates the marked dilatation of the upper of alimentary duct and the increased uptake in the pericardium

The patient group consisted of two male and seven female patients with an age range from 13 to 55 years (median age, 36 years). In our cases, the clinical manifestations were variable, including dyspnea (n = 6), a lack of symptoms (n = 2), and atrial fibrillation (n = 1). No cardiac tamponade occurred. The time from symptom onset to diagnosis was uncertain (ranging from several months to several years). All patients denied a history of trauma, congenital heart disease, thoracic surgery, tuberculosis or thoracic tumors. The patients underwent a physical examination, routine blood testing, standard chest radiography, electrocardiography and thoracic computed tomography (CT). Clinical examination showed elevated jugular venous pressure and distant heart sounds. The chest radiograph demonstrated enlargement of the cardiac silhouette, and echocardiography revealed pericardial effusion. Diagnostic pericardiocentesis was performed, and milky-colored pericardial fluid was aspirated from all patients. These findings, along with the high level of triglycerides in the pericardial fluid, sufficed to diagnose chylopericardium. All patients underwent extensive evaluation to find the cause of the chylopericardium. Routine laboratory tests demonstrated that there was no sign of a systemic inflammatory reaction. Cultures of the pericardial fluid for bacteria and tuberculosis were subsequently reported as negative, and cytologic examination showed no tumor cells. A chest CT scan did not reveal mediastinal neoplasm, mediastinal lymphadenopathy or other abnormalities that might have obstructed the thoracic duct. Lymphoscintigraphy was performed in all patients. Communication between the thoracic duct and the pericardial space was directly confirmed by lymphoscintigraphy in three patients. In three cases without communication, pericardial accumulation of technetium-99 m sulfur colloid and a possible site of leakage from the thoracic duct were confirmed. In the other 3 cases without communication, accumulation of technetium-99 m sulfur colloid alone was found (Figs. 1 and 2).

### Treatment

#### Non-surgical treatment

In our patients, non-surgical approaches were adopted for the treatment of chylopericardium: (1) pericardiocentesis, (2) pericardiostomy, and (3) dietary support with medium- or short-chain triglycerides and low-fat meals. Non-surgical treatment continued for approximately 2 to 3 weeks and was effective in 3 patients, in whom echocardiography showed no increase in pericardial effusion.

#### Surgical intervention

Because six patients did not respond adequately to repeated pericardiocentesis or continuous drainage of the pericardial effusion and a diet rich in medium-chain triglycerides (MCTs), surgical treatment was considered. The surgical procedure consisted of ligation of the thoracic duct just above the diaphragm, combined with the creation of a pericardial window. The surgical approach adopted was an open procedure in five cases (right posterolateral thoracotomy in four cases, midsternal thoracotomy in one case) and right video-assisted thoracoscopy in one case. The pericardial window was created anterior to the phrenic nerve to prevent the chylopericardium from giving rise to cardiac tamponade and further constrictive pericarditis in the future.

The patients tolerated the procedure well, and no patients died after the operation. A low-fat diet and initiation of MCTs were requested for no less than two weeks after the operation.

All of the patients remained stable postoperatively. The chest tube placed at the time of surgery was removed after the daily drainage decreased to below 100 ml, which occurred between the 3rd and 7th postoperative day. The patients were then discharged, with their chest X-ray showing a normal cardiac silhouette and an absence of pleural effusion.

However, one patient (case two in the Table 1) was readmitted because copious chylous fluid accumulated in the right pleural cavity two months later. It was suspected that the thoracic duct was not completely ligated. Therefore, a second operation to religate the thoracic duct was performed using the same approach. In this procedure, the thoracic duct was exposed between the esophagus, azygous vein and vertebra and was securely ligated above the diaphragm. After the second operation, the chylous accumulation in the pleural cavity was controlled. The daily drainage decreased to below 100 ml one week later and to 20 ml on the 22nd day postoperatively, after which the drain was removed. Once a chest X-ray and echocardiography showed an absence of pericardial and pleural effusion, the patient was discharged.

#### Follow-up

All patients were followed up from three months to nine years. There was no recurrence of pericardial effusion, pleural effusion, cardiac tamponade or constrictive pericarditis. Their chest X-ray showed a normal cardiac silhouette, or echocardiography showed a lack of pericardial effusion.

## Discussion

Chylopericardium is a rare clinical entity in which chylous fluid accumulates in the pericardial cavity. This entity results from thoracic duct disruption or obstruction, with both inadequate collateral drainage and reflux of chylous lymph through the lymphatics draining the heart and pericardium [3]. Secondary chylopericardium is frequently caused by disruption or obstruction of the thoracic duct as a result of thoracic or cardiac surgery, chest trauma, mediastinal neoplasms, mediastinal tuberculosis, or mediastinal radiotherapy, and it is occasionally a consequence of thrombosis of the subclavian vein.

Hasebrock was the first to describe the presence of 22.6 ml of chyle in the pericardial cavity [4], detected during the autopsy of a man who had died from asphyxia secondary to constriction and tracheal ulceration. The term “primary chylopericardium” was first used by Groves and Effler [5], who described a case of isolated accumulation of chyle in a 31-year-old woman who was found to have isolated, recurrent accumulation of chyle in the pericardium associated with mediastinal cystic hygroma. They used the term “primary idiopathic chylopericardium” in this first case report because no apparent etiologic mechanism was evident.

Primary idiopathic chylopericardium is a rare pathology, with very few cases reported to date. From Groves and Effler’s description in 1954 [5] until 2007, Silva reported that 114 cases of “primary” or “idiopathic” chylopericardium had been described in the literature [6]. We have found 14 new cases reported since 2007, with a total of 128 cases described up to November 2013. Herein, we described nine patients with this clinical entity at our hospital. To the best of our knowledge, this is the largest case series reported in English to date.

The etiology of chylopericardium is obscure, although in recent years, several reports have identified lymphatic leakage and communication with the pericardial sac, which are visualized by lymphangiography, combined CT and lymphangiography, or an intraoperative thoracic ductogram [7]. Damage to the thoracic duct valves, abnormal communication between the thoracic duct and the pericardial lymphatics or abnormally elevated pressure in the thoracic duct and increased permeability of the lymphatics may represent alternative mechanisms to explain the pathogenesis of chylopericardium.

Primary idiopathic chylopericardium occurs in all age groups and affects both sexes equally [8]. The clinical manifestations may vary from an absence of symptoms to signs of cardiac tamponade [9,10]. The most common presentations of patients with chylopericardium are a lack of symptoms, cough, dyspnea, and fatigue according to Mask et al. [11].

A chest radiograph showing an enlarged cardiac shadow is the first step in diagnosis. A cardiac ultrasound follows to elucidate the cause of cardiomegaly and to confirm the presence of pericardial fluid. The diagnosis requires an analysis of the fluid obtained. A milky fluid that contains fat, high levels of triglycerides and proteins and an elevated lymphocyte concentration confirms the diagnosis of chyle accumulation [11,12].

When the diagnosis of chylopericardium has been established, a search for the underlying cause should be performed. Exclusion of a history of trauma caused by thoracic surgery or blunt injury is needed. The introduction of subclavian venous catheters or episodes of vomiting or violent coughing should be noted [13]. Investigations specifically focused on detecting malignant disease, lymphoma, and tuberculosis should be conducted. A CT scan or MRI may then be performed to exclude lymphatic obstruction caused by a mass. Tuberculosis could be excluded using cultures and microscopic examination of the pericardial fluid. Lymphangiography may demonstrate the presence of communication between the pericardial sac and the lymphatic system in certain cases [7]. If no obvious cause can be established, the entity is labeled “primary idiopathic chylopericardium.”

In our cases, we excluded the common causes of chylopericardium. For this reason, we concluded that these patients should be diagnosed with primary idiopathic chylopericardium. One of our patients did have a history of treatment for tuberculosis, but there was no clinical or other evidence of pulmonary tuberculosis upon investigation.

The management of chylopericardium is centered on the prevention of the mechanical (cardiac tamponade or constructive pericarditis), metabolic and immunological consequences of chylopericardium and seeks to eliminate lymphatic fluid losses and to reduce recurrence. Initially, patients should be treated with non-surgical measures, such as (1) pericardiocentesis; (2) pericardial drainage; (3) dietary support with a low-fat diet and initiation of MCTs, which are absorbed via the portal vein, rather than via the lymphatic vessels, or even with total parenteral nutrition; and (4) pharmacological therapy with octreotide, which has been shown to reduce intestinal absorption of fats [14].

Non-surgical treatment of idiopathic chylopericardium is usually not satisfactory, and a failure rate of 57 to 60 % has been reported [1,15]. Rusca reported that medical therapy should not be continued for longer than two weeks [16]. If medical therapy proves to be ineffective, then surgical treatment should be considered, even in asymptomatic patients, to avoid subsequent progression to cardiac tamponade or constrictive pericarditis [17].

There are different approaches to the surgical treatment of chylopericardium: (1) pericardiectomy, (2) pericardial window formation, and (3) ligation of the thoracic duct above the level of the diaphragm. Pericardiectomy is performed to ensure complete drainage and to prevent later constrictive pericarditis. Pericardial window formation alone is simple but carries a high incidence of recurrence because it does not close the communication between the thoracic duct and the pericardial sac [1,15]. The necessity of thoracic duct ligation is well illustrated by a case reported by Musemeche and associates [18], wherein partial pericardiectomy and the creation of a pericardial window without thoracic duct ligation were followed by continued chylous leakage. After a second operation with ligation of the thoracic duct, the drainage abruptly ceased. Akamatsu et al. [1] reviewed 79 cases described in the literature up to 1992 and reported that 69 patients (87 %) were surgically treated, with 41 (52 %) undergoing ligation plus resection of the thoracic duct associated with pericardial window formation. According to these reports, thoracic duct ligation and pericardial window formation are believed to be the most effective procedures to prevent recurrence.

The thoracic duct, especially in the lower thoracic cavity, is accessible during surgery from either the right or the left side. Most researchers believe that the left-sided approach has certain disadvantages. In the lower thoracic cavity, the thoracic duct is located between the azygous vein and the descending aorta. The heart and the descending aorta hinder the exposure of the duct from the left side. It is easy to access the duct when entering from the right hemithorax.

Furrer and Nikolaos performed duct ligation and pericardial window formation by means of right-sided thoracoscopy [19,20]. The identification and ligation of the thoracic duct from a low position are no different from the methods used in traditional surgery. As thoracoscopic surgery is much less invasive than conventional open surgery and leaves minimal scarring, we recommend a right-sided thoracoscopic approach to the thoracic duct in the lower thoracic cavity.

For a patient who refuses surgical treatment but who exhibits continuous pericardial effusion, pericardio-peritoneal shunt placement [21] or thoracic duct embolization (TDE) [22] may be a simple and effective alternative.

As cardiac tamponade, constructive pericarditis or recurrence may occur, patients with chylopericardium should be regularly followed. In our cases, all patients were well, with no complaints, when they were followed up from three months to nine years. Thus, we believe that the outcomes of all of our patients were favorable, despite one patient having undergone two operations.

## Conclusions

In summary, primary idiopathic chylopericardium is a rare condition. It should be included in the differential diagnosis of a patient presenting with an enlarged cardiac silhouette. The patient should be evaluated rapidly to prevent the complications of this disease. We recommend a brief trial of medical therapy for no longer than three weeks, followed by thoracic duct ligation and pericardial window formation by right-sided thoracoscopy if this therapy fails. Dietary restriction needs to be continued following surgical therapy. All patients should be followed up for a long period of time.
